# Environmental dust effects on aluminum surfaces in humid air ambient

**DOI:** 10.1038/srep45999

**Published:** 2017-04-05

**Authors:** Bekir Sami Yilbas, Ghassan Hassan, Haider Ali, Nasser Al-Aqeeli

**Affiliations:** 1 Department of Mechanical Engineering, King Fahd University of Petroleum and Minerals (KFUPM), Dhahran 31261, Saudi Arabia; 2 Center of Research Excellence in Renewable Energy (CoRE-RE), King Fahd University of Petroleum and Minerals (KFUPM), Dhahran 31261, Saudi Arabia

## Abstract

Environmental dusts settle on surfaces and influence the performance of concentrated solar energy harvesting devices, such as aluminum troughs. The characteristics of environmental dust and the effects of mud formed from the dust particles as a result of water condensing in humid air conditions on an aluminum wafer surface are examined. The dissolution of alkaline and alkaline earth compounds in water condensate form a chemically active mud liquid with pH 8.2. Due to gravity, the mud liquid settles at the interface of the mud and the aluminum surface while forming locally scattered patches of liquid films. Once the mud liquid dries, adhesion work to remove the dry mud increases significantly. The mud liquid gives rise to the formation of pinholes and local pit sites on the aluminum surface. Morphological changes due to pit sites and residues of the dry mud on the aluminum surface lower the surface reflection after the removal of the dry mud from the surface. The characteristics of the aluminum surface can address the dust/mud-related limitations of reflective surfaces and may have implications for the reductions in the efficiencies of solar concentrated power systems.

Climate change gives rise to dust storms and the settlement of environmental dust particles on the surfaces of energy harvesting devices, such as solar concentrated troughs. The increasing frequency of dust storms, particularly in the Middle East, has made dust settlement one of the challenging problems in this region. The dust particles are of various shapes and densities, and their elemental compositions include silica, calcite, metallic oxides, alkaline metals and chlorine^1^. In some cases, the adhesion between the dust particles and settled surface remains strong, and external efforts are required to remove the particles from the settled surfaces. In general, the adhesion force between the dust particles and solid surface is influenced by the free energies of the solid surface and dust particles as well as the interfacial energy between the dust particles and the solid surface. Metallic surfaces have high surface free energies, which makes it difficult to remove the settled dust particles from the metallic surfaces. In addition, water molecules condense on the settled dust particles in humid air conditions, which in turn changes the physical and chemical properties of the dust particles^2^. Furthermore, alkaline and alkaline earth metals in dust particles dissolve in water condensate and form a chemically active liquid solution^3^. Due to gravity, the liquid solution flows through the solid surface and forms an interlayer between the dust particles and solid surface. Once the liquid solution layer dries, it forms crystalline structures at the interface of the dust particles and solid surface. These structures further increase the adhesion between the dust particles and the solid surface. Consequently, the effort required to remove the dust particles from the solid surface becomes significant and involves a high cost. Moreover, the liquid solution at the interface contains basic ions, which have an adverse effect on the solid surface integrity because ions chemically attack the solid surface, giving rise to corrosion on the surface prior to drying. Once the surface integrity of aluminum is damaged due to chemical attack by the liquid solution, the optical properties of the surface, such as its reflectivity and absorptivity, can be different even after the dried mud is removed from the surface. Aluminum is widely used in solar energy harvesting systems as reflecting surfaces, such as troughs, which improves the solar concentration by focusing solar radiation onto a thermal harvesting unit, such as a solar heat exchanger. However, environmental dust settlements and mud formation on the aluminum surface modify the optical characteristics of the aluminum trough surface while reducing the concentrated solar radiation in a solar harvesting system. Consequently, investigation of the adhesion of environmental dust particles and mud on aluminum surfaces as well as the long-term effects of such adhesion become essential.

Numerous research studies have been conducted to examine the characteristics of environmental dust and their effects on solar harvesting devices. Rifai *et al*.^4^ studied the mechanics of dust removal from a rotating disk in relation to self-cleaning applications of a PV protective cover. They found that the centrifugal force remained higher than the adhesion, friction, drag, lift, and gravitational forces in the area away from the rotational center. The dust particle size and rotational speed significantly influenced the rate of dust removal from the disk surface. Costa *et al*.^5^ investigated environmental dust soiling on solar energy systems and presented a review on solar-device soiling and mitigation approaches published over the past five years. Sueto *et al*.^6^ studied the suppression of dust adhesion on a concentrator photovoltaic module using an anti-soiling photocatalytic coating. They found that the presence of electrostatic charges on the surface was the main factor for the adhesion of sand, and such charges could be suppressed by an anti-soiling photocatalytic layer. Javed *et al*.^7^ performed a dust characterization study in the Doha region of the Middle East and demonstrated that calcium was the most abundant element in the accumulated dust, followed by silicon, iron, magnesium and aluminum. Calcite, dolomite, and quartz were also dominant minerals in the accumulated dust, with gypsum being a minor component. Dust collected after dust-storm events had higher proportions of halite and quartz contents than dust collected on days without dust storms, which depended on the direction of the wind. Mehmood *et al*.^8^ performed a characterization study of the environmental dust collected from PV modules in the northern area of Saudi Arabia and found that the adhesion force remained high after removing the dry mud from the glass surface because of the dry mud solution located between the mud and the glass surface. Tanesab *et al*.^9^ examined the effect of dust on the performance of PV modules under harsh environmental conditions and found that the output power of the PV modules decreased by 19% to 33%, where the degradation was largely due to the dust settlement and some non-dust related factors, such as corrosion. by Klugmann-Radziemska^10^ investigated the degradation of the electrical performance of a crystalline photovoltaic module subjected to dust deposition in northern Poland and found that the electric performance of the PV module degraded due to the dust deposition, which was closely related to the tilt angle of the module, exposure period, site climate conditions, wind movement, and dust properties. Elminir *et al*.^11^ investigated the influence of dust particles on the transparent cover of solar collectors and found that a reduction in the glass normal transmittance was strongly dependent on the dust deposition density, plate tilt angle, and orientation of the surface with respect to the dominant wind direction. Paudyal and Shakya^12^ investigated the effects of dust on the efficiency of PV panels and presented a review of the degradation of a PV panel performance due to dust-induced physical damages, such as attenuation of the incident solar radiation and increases in the temperature of the PV module. Christo^13^ performed a numerical study of wind and dust patterns around a full-scale paraboloidal solar dish and found that vortex shedding occurred when the pitch angle of the solar panels was high, with a strong oscillation flow extending downstream of the dish. Furthermore, the dust settlement on the panel surface was dependent on the flow pattern at the panel surface. Shehri *et al*.^14^ investigated the impact of dust deposition and brush-based dry cleaning on the glass transmittance of the PV modules. They demonstrated that the process of brushing dusty samples improved the transmittance compared to the un-brushed state. However, the cleaning efficiency of the nylon brushes was lower than that of water and delicate wipers. In addition, there were some changes in the surfaces of the glass samples after brushing.

Dust adhesion on surfaces is critical in terms of removing the dust from the surface and the aftereffects of such dust on the optical properties of the surface. Although several studies have discussed dust adhesion on surfaces, they mainly focused on preventing and minimizing dust adhesion onto the surfaces of optically transparent wafers^1^,^3^. Dust adhesion on highly reflective metallic surfaces pertinent to solar thermal applications, such as aluminum trough surfaces, in humid environments and its effects on the surface properties were not investigated. Therefore, this study investigates environmental dust and mud adhesion on aluminum surfaces and the aftereffects of dust and mud on the surface characteristics of the aluminum. The elemental composition and size distribution of the environmental dust particles are estimated. The mud formed from the dust particles, mimicking water condensation on the dust surfaces, is examined specifically. The adhesion force required to remove the dust particles and dry mud from the aluminum surfaces is measured using a microtribometer. The morphological changes of the aluminum surface after the mud removal are analyzed by scanning electron and optical microscopes. The optical properties of the aluminum surface after the mud removal are assessed using a UV-visible spectrometer. The elemental compositions of the residues of the dust particles and dry mud after removal from the aluminum surface are analyzed using X-ray diffraction and energy dispersive spectroscopy.

## Experimental

**Figure 1:**
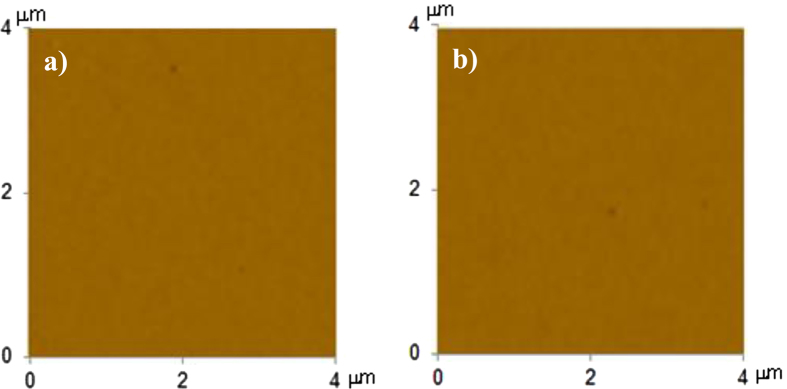
AFM images of aluminum surface: (**a**) prior to water jet cleaning of as received surface, (**b**) after water jet cleaned as received surface.

Aluminum wafers with a thickness of 3 mm and high optical reflectivity were used as the workpieces. The aluminum wafers were cleaned ultrasonically and chemically prior to the experiments. The mud was formed from a mixture of environmental dust particles and desalinated water while water condensation on the dust particles in humid air conditions was mimicked. The dust particles were collected from the local environment in the Dammam area of Saudi Arabia. The dust particles that had accumulated on the solar trough surfaces over a two-week period were removed using soft brushes with fine fibers. The brush was used only once, and a new brush was used for the dust removal from the second trough surface. The dust particles brushed from the trough surfaces were then collected in containers, which were sealed immediately after the collection of the dust particles to protect the collected dust particles from the environmental humidity and other contaminants. Using the collected dust particles in the sealed containers, a layer with a thickness of 300 μm was formed on the aluminum wafer surface. A dust layer with a thickness of 300 μm resembles the actual dust accumulation on surfaces in an open environment over a two-week period after a regular sand storm. To simulate the influence of the high humidity of the environment on the accumulated (settled at the surface) dust particles, initial tests were conducted to measure the amount of water vapor absorbed by the dust particles during the condensation of water vapor in a local humid environment over a six-hour period. The tests results showed that the volume of condensate was nearly equivalent to the volume of the dust particles after over six hours. To resemble actual water condensation on the layer of dust particles in a humid air environment, desalinated water having the same volume as the dust layer was dispensed gradually onto the dust layer. The dispensed water with the same volume as the dust particles on the aluminum surface was left to dry in a controlled cell without mechanical mixing; this gave rise to the natural formation of dry mud on the aluminum surface. Because the typical average day temperature is approximately 70 °C under the sun on most humid days during summer in Dammam, the temperature was set accordingly to 70 °C in the controlled cell during the drying period of the mud. The duration of the mud drying was set to 8 hours from the start of the drying process. Once the mud dried on the aluminum wafer surface, the workpieces with the dry mud were tested for adhesion work and friction coefficient measurements. In order to assess the strongly adhering of dry mud regions onto the aluminum surface, upon completion of the adhesion and friction tests, the dry mud layer was removed from the aluminum surface by a pressurized desalinated water jet with a diameter of 2 mm and a velocity of 2 m/s. The desalinated water jet does not have an abrasive effect during the dry mud removing from the surface because of the low impact pressure of the water jet onto the surface, which is in the order of 2 kPa gage. However, the jet velocity and the impact pressure of the water jet at the solid surface for the abrasive water jet processing is in the order of 350 m/s and 70 × 10^3^ kPa^15^. Therefore, the erosion of the water jet used in the dry mud cleaning is negligibly small. In order to examine the effect of desalinated water jet of 2 m/s velocity on the aluminum surface, jet impingement tests are carried out on the aluminum surface for 60 minutes. AFM micro images reveal that no erosion pattern is observed at the surface after the jet impingement tests of the as received aluminum surface (Fig. (1a) and (b). The water jet assisting the cleaning process was continued for 20 minutes for each workpiece surface. Finally, analytical tools were used to assess the morphology and optical characteristics of the aluminum surfaces with the dry mud removed.

The dust particles and dry mud on the aluminum surface were characterized using analytical tools. SEM and EDS examinations were performed using a JEOL 6460 electron microscope, and XRD analysis was peformed using a Bruker D8 Advanced having Cu–Kα radiation. A typical setting of the XRD was 40 kV, 30 mA and a scanning angle (2θ) that ranged from 20° to 90°.

Fourier transform infrared spectroscopy (Nicolet Nexus 670 FTIR) was used on the aluminum surfaces from which dry mud had been removed. A UV-VIS Spectrophotometer UV-2600 (Shimadzu) was used to measure the reflectance of the aluminum surfaces prior to and after the dry mud removal by the water jet.

Micro-scratch tests were performed to measure the adhesion and friction work for the dry mud removal from the aluminum surfaces. The standard test method stated in the ASTM D7027-05 standard was adhered to during the micro-scratch tests, and a linear micro-scratch tester (MCTX-S/N: 01-04300) was used for the scratch measurements. During the experiments, the equipment was set at a contact load of 0.03 N and an end load of 5 N. The total length for the scratch tests was 5 mm, and the scanning speed was maintained at 5 mm/min with a loading rate of 5 N/s.

## Results and Discussion

The effects of environmental dust and mud formed from dust particles on an aluminum surface while mimicking water condensation on the dust particles in a humid environment are examined. The dust particles are analyzed in terms of their size and elemental composition. The morphological changes on the aluminum surface after the dust and mud removal are examined using analytical tools. The adhesion work of removing the dust particles and dry mud from the aluminum surface is determined from mirotribometric data. The optical characteristics of the aluminum surface prior to and after mud removal are measured. The dust and mud residues after their removal from the surface are analyzed using X-ray diffractogram and energy dispersive and Fourier transform infrared spectroscopies.

**Figure 2:**
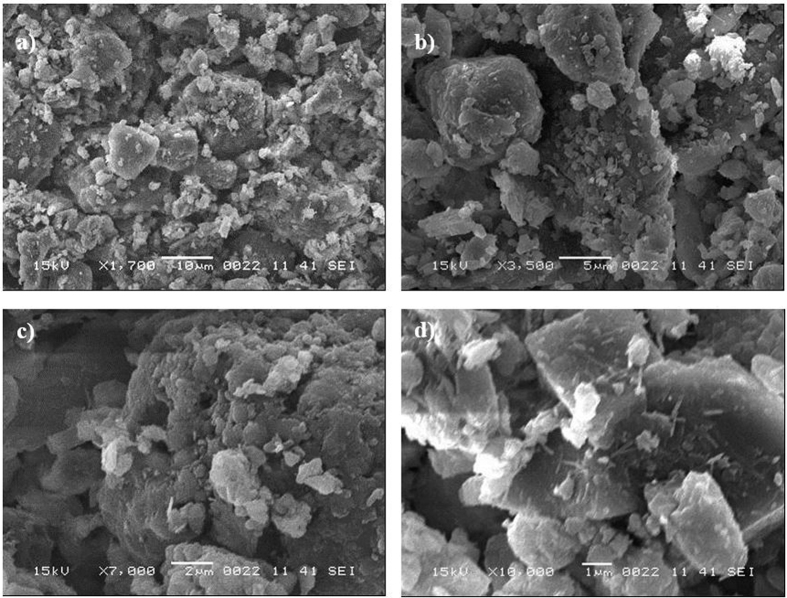
SEM micrographs of dust particles: (**a**) particles of different sizes, (**b**) particles with different shapes, (**c**) small particles attached to large particles, and (**d**) charged particles (bright color) on large dust particles.

**Figure 3:**
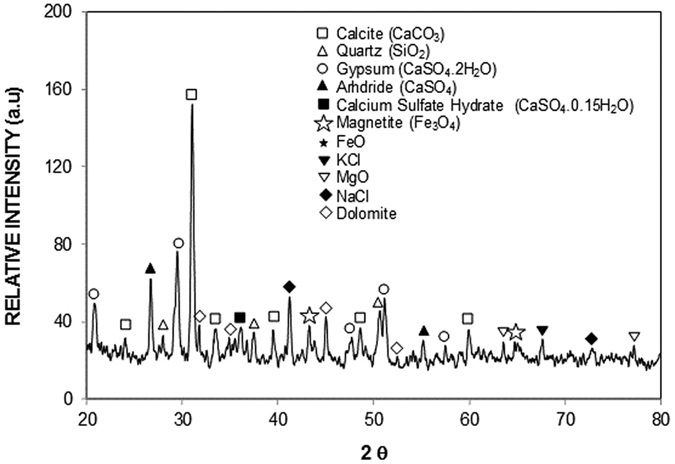
X-ray diffractogram of dust particles collected in the Dammam area of the Kingdom of Saudi Arabia.

**Figure 4:**
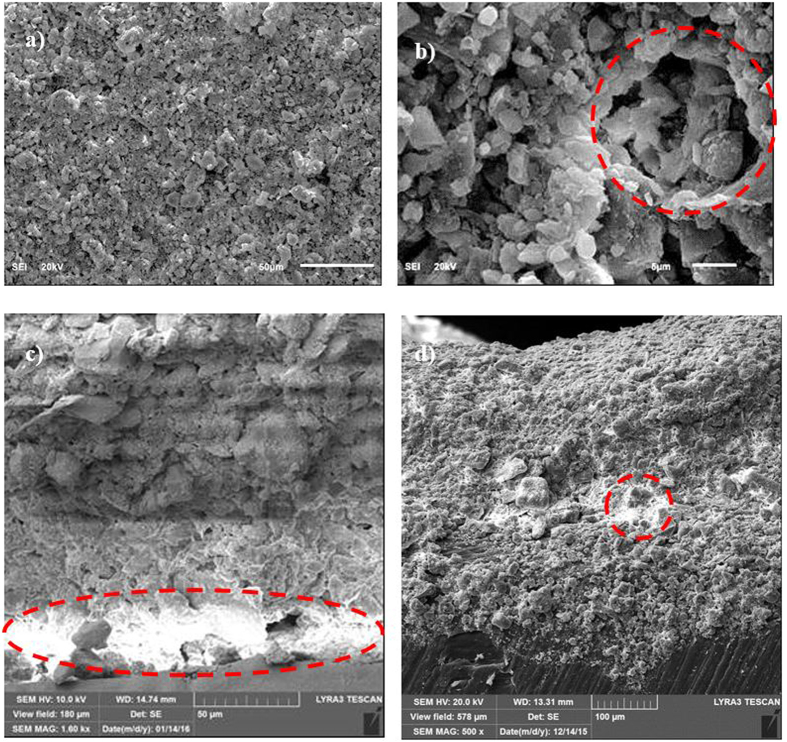
SEM micrographs of the dry mud top surface and cross-section: (**a**) top surface, (**b**) pore-like structures on the dry mud top surface, (**c**) dry mud cross-section. The dotted circle shows the dried mud liquid at the interface of the dry mud and solid surface. (**d**) Dried mud solution in a small cavity in dry mud. The dotted circle indicates the mud liquid captured in the small cavity inside the mud while forming dried mud liquid after mud drying.

**Figure 5:**
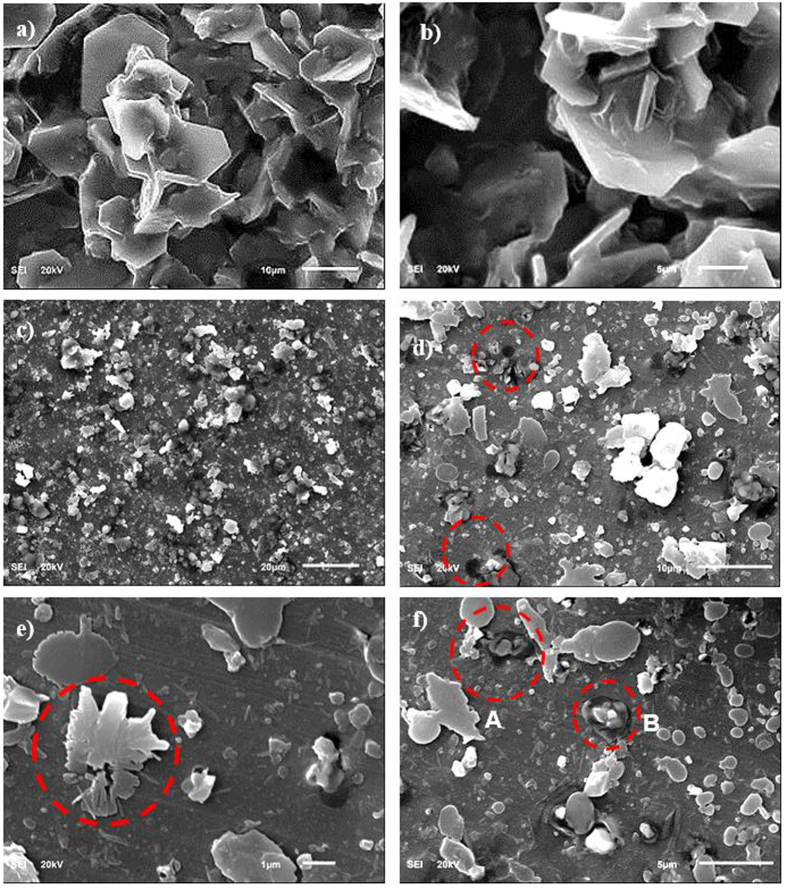
SEM micrographs of the dried mud solution and dry mud-removed aluminum surface: (**a**) crystals of the dried mud solution, (**b**) shape of the crystals formed, (**c**) dry mud-removed surface by a water jet, (**d**) pinholes formed on the aluminum surface after dry mud removal. The dotted circle indicates pinholes on the aluminum surface. (**e**) Reforming of the dried mud liquid crystals on the dry mud-removed surface, and (**f**) small pit sites on the aluminum surface after the dry mud removal.

Figure (2) shows the SEM micrographs of the dust particles, dry mud surface, and dry mud cross-section. The dust particles have different sizes and irregular shapes (Fig. (2a) and (b)); however, the small dust particles attach to the surfaces of the large dust particles (Fig. (2c) and (d)), which can be associated with the charges of the small size particles. In this case, the attachment of ionic compounds to small dust particles (<0.5 μm) can occur because small dust particles remain in the atmosphere for prolonged times in regions near the seacoast and interact with solar radiation for long durations^2^. Dust particles have various shapes, with an average size of 1.2 μm, and they aggregate to form small size clusters with diameters of 3 to 4 μm. The shape of the particles may be classified after considering the aspect ratio and shape factor^16^. The shape factor is related to the perimeter and cross-sectional area of the particles in the form of 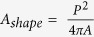, where *A* is the cross-sectional area and *P* is the perimeter of the dust particle^17^. The shape factor is an inverse of the particle circularity, which is associated with the complexity of the particle, where a shape factor of unity corresponds to a perfect circle. The aspect ratio is associated with the projection length and cross-sectional area of the dust particles, i.e., the aspect ratio is 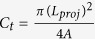, where *A* is the cross-sectional area and *L*_*proj*_ is the longest projection length of the dust particle^17^. The aspect ratio is associated with the particle roundness, which approximately characterizes the ratio of the major axis to the minor axis of the ellipsoid best fit to the particle. Moreover, for circular dust particles, the diameter of the circle and equivalent area are considered in calculations. However, for non-circular dust particles, an ellipse is considered, and the longest projection is assumed to be the major axis preserving the cross-sectional area of the dust particle. The findings revealed that there is no linear relation between the shape factor and aspect ratio and that both ratios change with varying dust particle sizes, i.e., the shape factor and aspect ratio increase with increasingly large dust particles; however, the aspect ratio increases to a lesser extent than the shape factor. The median shape factor is on the order of 3, whereas the aspect ratio remains at approximately 2.1 for particles >2.4 μm in size. However, the shape factor becomes close to unity for small particles, particularly for those with round shapes. The elemental composition of the dust particles varies irrespective of their shapes and sizes, and this variation is typically small. Table 1 provides the typical elemental composition of dust particles (wt%) obtained from the energy spectroscopy (EDS) data. In general, the quadrangular-shaped dust particles are rich in sodium and chlorine, and the aggregated small particles are rich in calcium and oxygen. The flat-shaped particles are rich in calcium and silicon. Elemental crystals and compounds are observed in Fig. (3), in which the X-ray diffractograms of dust particles are shown. The peaks of potassium, sodium, calcium, sulfur, chlorine, and iron are visible. However, the iron peak is coincident with the aluminum and silicon peaks. The presence of alkaline metal peaks (sodium and potassium) are related to the sea salt due to the local regional effect because the dust particles were collected near the Arabian Gulf. The stoichiometric molar ratio for NaCl is not satisfied in the EDS data (Table 1); therefore, the presence of NaCl is not in a crystal form in the dust particles but in a dissolved form. The presence of sulfur in the EDS data is related to the anhydrite or gypsum component (CaSO_4_). In addition, the iron is most likely related to clay-aggregated hematite (Fe_2_O_3_). In the case of the dry mud surface, the elemental composition remains similar to those of the loose dust particles (Table 1). The SEM micrographs of the topology of the dry mud surface are shown in Fig. (4a) and (b). The pore-like structures are formed on the mud surface because of the dissolution of some dust compounds, which flow toward the aluminum surface due to gravity. In this case, the elemental concentrations of alkaline metals (Na, K) and chlorine are reduced at the mud surface (Table 1) due to the dissolution of alkaline metals and chlorine in water in the wet mud while forming a mud liquid containing alkaline metals and chlorine. However, the mud liquid flows through the porous channels inside the mud and accumulates on the aluminum surface under gravitational force. To test the hypothesis of the dissolution of alkaline earth metals and chlorine in water, the dust particles are mixed with water at a 1/10 volume ratio (dust particles/water) for six hours. The pH of the resulting liquid solution is measured, and the solution is tested using inductively coupled plasma mass spectrometry (ICP). The pH increases rapidly to 8.2 within one hour, and similar data have been reported in previous studies^1^,^3^. The ICP data, which is given in Table 2, reveal that the alkaline (Na, K) and earth alkaline (Ca, Mg) metal compounds dissolve in a liquid solution. Consequently, the Na, K, and Cl concentrations in the surface region of the mud decrease significantly. The settled mud liquid forms a thin layer at the interface of the mud and aluminum surface. After the mud dries, the residues of the dried mud liquid at the interface form a thin layer of dried mud solution. In addition, the pores and voids are observed in the SEM micrograph of the dry mud cross-section (Fig. (4c) and (d)). Moreover, some mud liquids, captured in cavities formed between the dust particles in the mud, dry out and form a bright region in the micrograph (Fig. (4d)). The EDS analysis reveals that the dry mud solution in the cavities displays similar elemental characteristics as that at the interface between the aluminum and mud (Table 1). The morphology of the dry mud solution on the aluminum surface is also examined to assess the structural integrity of the mud solution upon drying. Figure (5a) and (b) show the SEM micrograph of the dried mud solution. Some small crystals are formed at the surface; however, these crystals are considerably smaller than those reported in previous studies^1^,^3^. This smaller size is attributed to the heat transfer from the mud liquid to the solid surface during drying. Because the thermal diffusivity of aluminum is considerably larger than those of glass and polycarbonate, as used in the previous studies^1^,^3^, high cooling rates decreases the crystal size at the surface during the drying period.

**Figure 6:**
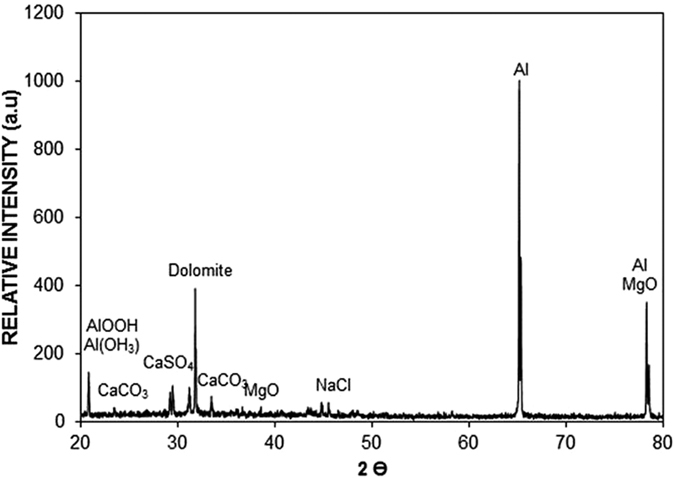
X-ray diffractogram of the dried mud solution on the aluminum surface.

**Figure 7:**
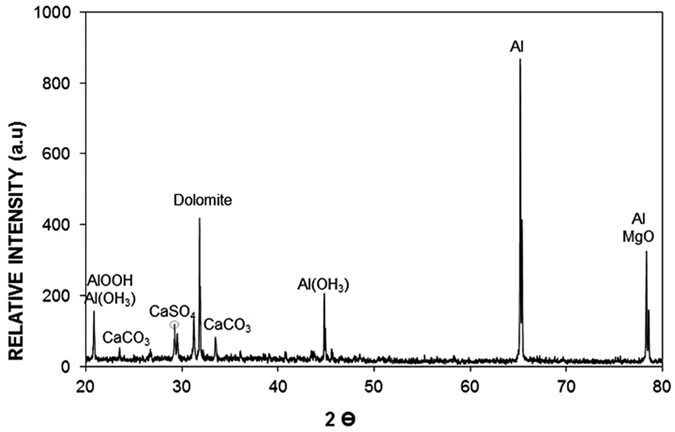
X-ray diffractogram of the dry mud-removed aluminum surface.

**Figure 8:**
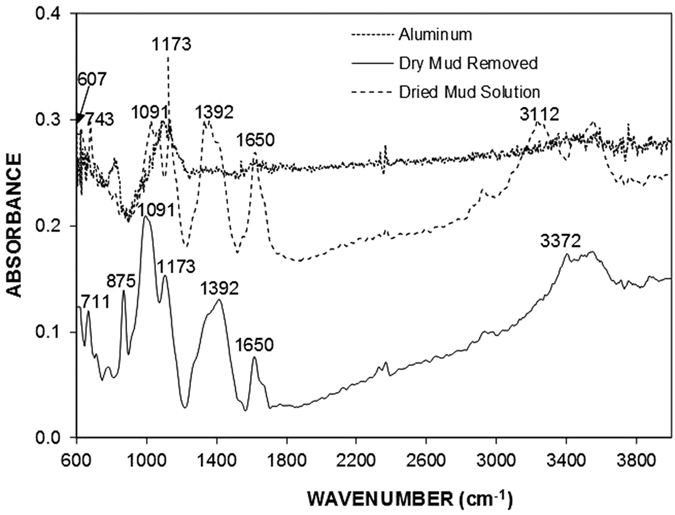
FTIR absorbance spectra of the dry mud-removed surface, dried mud solution, and as-received aluminum surface.

To assess the aftereffects of the dry mud on the aluminum surface, a water jet is used to remove the dry mud from the surface. In this case, a water jet with a diameter of 2 mm is maintained at a jet velocity of 2 m/s, and the dry mud removal and surface cleaning is continued for 20 minutes. The SEM micrographs of the dry mud-removed surfaces are shown in Fig. (5c)–(f). Mud residues of various sizes are scattered on the aluminum surface. The presence of the mud residues indicates the strong adhesion of the dry mud on the aluminum surface. Hence, the pinning of the dry mud residues occurs despite the use of the water jet for the dry mud removal from the aluminum surface. The strong adhesion of the dry mud residues on the aluminum surface is associated with the interfacial bonding on the aluminum surface because of the dry mud solution. Close examination of the surface reveals that fine-sized crystals of mud residues form on the surface after the dry mud removal and cleaning of the aluminum surface by the water jet. This indicates that the dried mud solution dissolves in the water on the aluminum surface during the water jet cleaning, and upon drying, they form crystal structures on the surface. The dry mud residues are mainly composed of alkaline (Na) and alkaline earth (Ca, Mg) metal compounds, as can be observed from the EDS data (Table 1). The elemental composition of the dry mud solution on the aluminum surface is quantified using energy dispersive spectroscopy to clarify the contribution of the dry mud solution to the dry mud residues. The elemental composition of the dry mud solution is also given in Table 1 for comparison. The elemental composition of the dry mud solution on the aluminum surface mainly consists of alkaline and alkaline earth metals. Therefore, the crystal structures formed beside the dry mud residues on the aluminum surface are attributed to the crystallization of the dried mud solution after removing the dry mud from the aluminum surface. This situation is also observed in Fig. (6), in which the X-ray diffractograms of the dried mud solution and dry mud residues are shown after removing the dry mud from the aluminum surface. The presence of NaCl, CaCO_3_, MgO, and CaSO_4_ peaks for both diffractograms reveals the residues of the dry mud solution on the dry mud-removed aluminum surface. However, locally scattered pinhole-like cavities are observed on the dry mud-removed aluminum surface (Fig. (5d)). The formation of pinhole-like structures is associated with the adsorption of chloride ions (Cl^−^) on the aluminum surface during the accumulation of the liquid mud solution, which contains ionic compounds, including chlorine (Table 1). The penetration of the aluminum surface by chloride ions results in Cl^−^ assisted dissolution beneath the surface, which can in turn initiate the pinhole formation at the surface due to the localized dissolution of aluminum at the metal/oxide interface in the consecutive one-electron transfer reactions. In addition, oxidation of the aluminum surface can give rise to the formation of hydroxyl groups covering the surface under the aqueous mud solution, and the hydroxyl groups may remain undissociated. Because the pH of the mud solution is high (pH = 8.2), the surface acquires a positive charge, i.e.: −OH_surf_ + OH^−^ ↔ −O^−^_surf_ + H_2_O^18^. Therefore, the liquid mud solution interacts with the aluminum surfaces to form hydroxyl groups, and the component due to OH^−^ increases as the exposure of the surface to the liquid mud solution increases. This results in the formation of the partially hydroxylated aluminum oxide layer, such as AlOOH^19^. This situation is also seen from the X-ray diffractogram of the aluminum surface after the dry mud removal from the surface (Fig. (7)). Figure (8) shows the Fourier transform infrared spectroscopy data for the aluminum surface after the dry mud removal. The presence of hydroxylated aluminum on the dry mud-removed surface is evident from the intense band at 1091 cm^−1^ and other intensities at 1392 cm^−1^ and 1650 cm^−1^. The band at 1091 cm^−1^ is attributed to the Al-OH hydroxyl bending mode in aluminum oxy-hydroxides^20^,^21^. The large band between 3260 and 3680 cm^−1^ is associated with the stretching vibration of OH in the hydroxyl groups. In this case, the intensity peaks at 3372 and 3112 cm^−1^ correspond to the (Al)O-H stretching vibrations^22^. In addition, the band at the shoulder at 1173 cm^−1^ is related to the Al-O-H mode of γ-AlOOH. The intensity peaks at 743 and 607 cm^−1^ describe the vibration mode of Al-O. The absorption peaks at 711 and 875 cm^−1^ are related to the in-plane and out-of-plane bending vibration of carbonate ions, respectively. The presence of the peaks of carbonate ions is associated with the CaCO_3_ residues on the surface after the dry mud removal.

**Figure 9:**
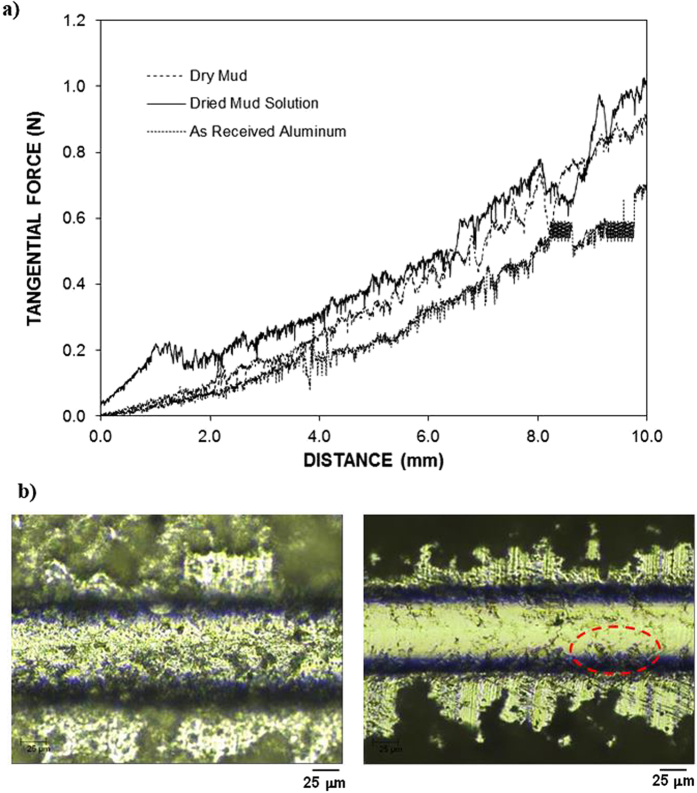
(**a**) Tangential force variation along the scratch length and (**b**) scratch marks on the aluminum surface. The dotted circle indicates the dry mud residues along the scratch length.

**Figure 10:**
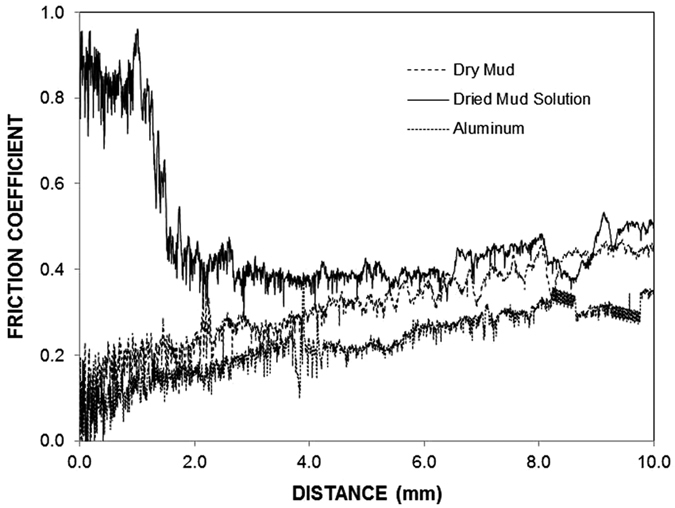
Dynamic friction coefficient of the aluminum surface when dry mud and dried mud solution are present on the surface. The friction coefficient of aluminum is also shown for comparison.

**Figure 11:**
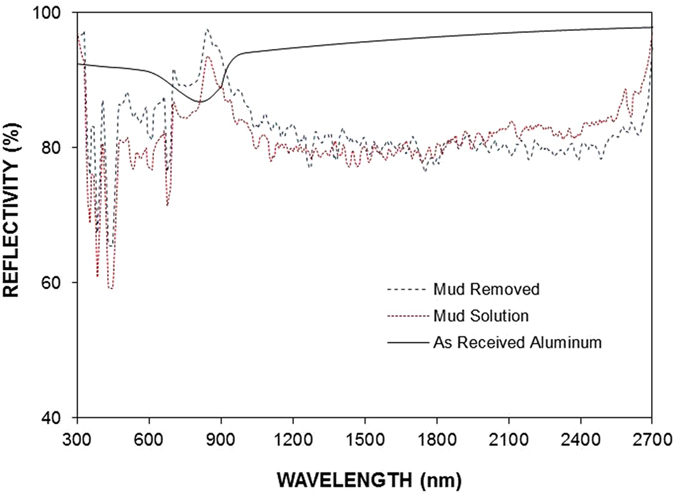
Reflectance of the UV-visible spectrum for the dry mud-removed aluminum surface, the aluminum surface with the dried mud solution, and the as-received aluminum surface.

Figure (9a) shows the tangential force along the length of the dry mud, dried mud solution, and as-received aluminum surfaces, and Fig. (9b) shows the scratch marks left after the micro-scratch tests. The area under the tangential force is the work required to remove the dry mud and dried mud solution and overcome the friction on the aluminum surface. The tangential force required to remove the dried mud solution from the aluminum surface remains the highest, followed by those required for dry mud and overcoming friction. The tangential force involves adhesion and frictional forces, which are related to the dry mud and dried mud liquid adhesion on the surface. However, several forces contribute to the adhesion of the dry mud on the aluminum surface, such as van der Waals and electrostatic forces^23^. Because the liquid mud solution interacts with aluminum surfaces to form hydroxyl groups, the compounds formed at the surface during the chemical attack give rise to the formation of chemical bonding at the surface. Consequently, the increase in the tangential force for the dried mud solution is related to the strong adhesion of the dried mud solution on the aluminum surface. Because the dried mud solution forms a layer at the interface of the dry mud and aluminum surface, the adhesion force between the dry mud and aluminum surface also remains high. However, the local distribution of the dried mud liquid at the interface becomes critical for dry mud adhesion on the aluminum surface. In this case, dry mud adhesion remains high for those areas where the dried mud solution forms an interlayer between the dry mud and aluminum surface. This gives rise to some small high frequency oscillations in the tangential force along the scratching direction on the aluminum surface, which appears as noise long the curve (Fig. (9a)). The strong attachment of the dry mud on the aluminum surface results in dry mud residues along the scratch mark. This situation can be observed from the image of the scratch mark (Fig. (9b)). Hence, the presence of the dry mud residues on the aluminum surface after the dry mud removal by the water jet (Fig. (5c)) is attributed to the strong bonding of the dry mud on the aluminum surface due to an interfacial layer of the dried mud solution. In the case of the dried mud solution, some striations are observed along the scratch marks, which indicate the strong attachment of the indenter tip on the dried mud surface and the scale buildup around the indenter tip. The adhesion work determined from the difference between the tangential and frictional forces is shown in Table 3. The adhesion work required to remove the dried mud solution from the aluminum surface is nearly 20% more than that of the dry mud. Consequently, the dried mud solution formed at the interface between the dry mud and aluminum surface contributes significantly to the adhesion work required to remove the dry mud from the aluminum surface. Figure (10) shows the friction coefficient of the aluminum surface prior to and after the dry mud removal. The friction coefficient due to the dry mud solution is shown for comparison. The friction coefficient remains the highest for the dried mud solution, followed by the dry mud-removed and as-received aluminum surfaces. The attainment of a high friction coefficient for the dry mud solution is attributed to the large adhesion force between the indent tip and surface. In addition, the presence of the crystals contributes to the friction coefficient. In the case of the dry mud-removed surface, the friction coefficient remains higher than that corresponding to the as-received aluminum surface. Thus, the mud residues at the surface increase the friction coefficient, but this increase is not substantial. Some small high frequency oscillations is related to the adhesion and releasing of the pin during scratching as similar to that explained for Fig. (9a). Figure (11) shows the reflectance characteristics of the aluminum surface prior to and after the dry mud removal from the surface. The surface reflectance decreases significantly after the dry mud removal from the aluminum surface due to the residues of the dry mud and dried mud solution on the aluminum surface, which cause absorption, diffraction, and scattering of the incident UV-visible radiation. The reflectance decreases particularly for the wavelengths between 900 and 2,700 nm. The decrease in the reflectance is associated with mud residues on the surface after removing the dry mud from the surface by a water jet. In this case, the mud residues act as a diffusive surface and cause scattering of the incident UV-visible spectrum on the surface. In addition, the reflectance of the incident UV-visible radiation decreases significantly when the surface is covered by the dry mud solution. Consequently, the mud formed on the surface of the aluminum wafer results in a significant reduction of the reflection of the incident UV-visible radiation despite its removal from the aluminum surface.

## Conclusion

The influence of the dust and mud formed from dust particles on the surface characteristics of an aluminum wafer is examined. The dust particles are collected from the Dammam area of Saudi Arabia. Water condensate on the dust particles in a humid air environment is simulated by mimicking the environmental conditions. The wet mud formed on the aluminum surface is then dried, resembling the environmental conditions prior to the tests. The collected dust particles are characterized using analytical tools, including scanning electron and optical microscopes, energy dispersive spectroscopy, and X-ray diffraction. The size distribution of the dust particles is analyzed, and the dust particle shape is assessed through the shape factor (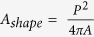, where *A* is the cross-sectional area and *P* is the perimeter of the dust particle) and aspect ratio 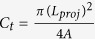, where *A* is the cross-sectional area and *L*_*proj*_ is the longest projection length of the dust). The elemental quantification of the dissolved dust compounds in water is assessed using inductively coupled plasma mass spectrometry. The adhesion work required to remove the dry mud from the aluminum surface is determined using a microtribometer. Then, the dry mud formed on the aluminum surface is removed by a water jet, and the residues of the dry mud on the surface are analyzed. In general, the dust particles have various shapes, with an average size of 1.2 μm. The small size dust particles attach to the large particles because of the electrostatic force. Because the dust particles possess alkaline (Na. K) and alkaline earth (Ca, Mg) metals, these compounds dissolve in water condensate and form the wet mud and wet mud solution. Due to gravity, the wet mud solution flows through dust particles and settles at the interface of the wet mud and aluminum surface while forming locally scattered small layers of liquid film. The liquid mud solution is chemically active, increases the water pH to 8.2 and gives rise to the formation of pinholes and extremely shallow pit sites on the aluminum surface. Upon drying, the liquid mud solution forms small crystals at the surface and increases the adhesion between the dry mud and aluminum surface. The work of adhesion on the aluminum surface remains high for the dry mud solution, followed by that required for the dry mud. The mud residues are observed after the removal of the dry mud from the aluminum surface by the water jet. The strong adhesion between the dry mud and aluminum surface is a result of the presence of the dried mud liquid at the interface. The mud residues on the surface alter the adhesion force and reflectance of the aluminum surface. The UV optical reflectance of the aluminum surface is significantly affected by the dry mud and mud residues. The surface reflectivity is reduced by over 22% when the dry mud is removed from the surface by a water jet. Consequently, after the dry mud removal, the characteristics of the aluminum surface could not be regained due to the aftereffects of the mud formed on the surface, such as pitting sites and pinhole formation on the surface. The present work provides useful information regarding dust and mud adhesion on highly reflective surfaces, such as aluminum, and demonstrates that the optical properties of the aluminum surface cannot be regained despite removal of the mud and the dust from the surface.

## Additional Information

**How to cite this article**: Sami Yilbas, B. *et al*. Environmental dust effects on aluminum surfaces in humid air ambient. *Sci. Rep.* **7**, 45999; doi: 10.1038/srep45999 (2017).

**Publisher's note:** Springer Nature remains neutral with regard to jurisdictional claims in published maps and institutional affiliations.

## Figures and Tables

**Table 1:** Elemental composition of the dust (wt%).

	Si	Ca	Na	S	Mg	K	Fe	Cl	O
Dust	11.8	8.1	3.5	2.6	2.4	1.1	1.2	0.8	Balance
Mud Solution	—	6.2	3.4	—	1.6	1.1	—	0.8	Balance
Dry Mud	12.3	6.3	0.1	2.5	1.7	0.1	1.2	0.2	Balance
Mud Residues	8.2	5.2	1.8	0.8	1.9	0.8	0.4	0.7	Balance

**Table 2:** ICP data for the mud solution after 8 hours of dissolution of the dust particles in desalinated water.

Ca	Na	Mg	K	Fe	Cl
268500	52000	66200	31000	1500	32500

**Table 3:** Frictional and adhesion work obtained from the tangential force.

	Frictional Work (mJ)	Adhesion Work (mJ)
As-Received Surface	1.351	—
Dried Mud Solution	—	2.211
Dry Mud	—	1.861
